# Immunologic comparison of alpha-gal sensitized and non-sensitized groups from a community based sample

**DOI:** 10.3389/fimmu.2026.1824819

**Published:** 2026-09-30

**Authors:** Emily Owens Pickle, Kyndall Dye-Braumuller, Kia Zellars, Stella C.W. Self, Scott Commins, Melissa S. Nolan

**Affiliations:** 1 Department of Epidemiology and Biostatistics, Arnold School of Public Health, University of South Carolina, Columbia, SC, United States; 2 Institute for Infectious Disease Translational Research, University of South Carolina, Columbia, SC, United States; 3 Allergy & Immunology School of Medicine, University of North Carolina, Chapel Hill, NC, United States

**Keywords:** alpha-gal, alpha-gal allergy, innate immunity, pro-inflamatory cytokines, Th2 polarization

## Abstract

**Introduction:**

Alpha-gal syndrome is delayed, IgE-mediated hypersensitivity reaction to galactose-α-1, 3-galactose (alpha-gal), a carbohydrate epitope found in non-primate mammals. Sensitization is typically triggered by tick bites, with some sensitized individuals progressing to severe allergic reactions to red meat and other alpha-gal containing substances. Despite increasing clinical recognition, the immunologic mechanisms underlying alpha-gal sensitization remain poorly defined. Therefore, this article aimed to identify whether there are distinct immunologic differences between sensitized and non-sensitized groups sampled from a well-characterized, community-based cohort.

**Methods:**

Participants were matched by age, sex, and race/ethnicity then classified as sensitized (AG+) or non-sensitized (AG−) by laboratory criteria. Demographics, health history, and target concentration between the groups were compared using descriptive statistics, Wilcoxon tests, and multivariable logistic modeling.

**Results:**

AG+ participants (n=50) showed elevated concentrations of innate immune markers including CD163, Chitinase-3-like 1, gp130, and MMP2 (all p < 0.0001), and reduced levels of regulatory cytokines including IL-10, IL-12p40, IL-35, LIGHT, and interferons IFNα2 and IFNβ. Logistic regression identified three independent cytokine predictors of alpha-gal sensitization: IL-1α (OR = 2.75, 95% CI: 1.40–5.41, p = 0.004), IL-35 (OR = 0.22, 95% CI: 0.10–0.49, p < 0.001) and LIGHT (OR = 0.19, 95% CI: 0.05–0.75, p = 0.02).

**Discussion:**

Our findings showed distinct differences between AG+ and AG- groups, marked by increased pro-inflammatory signaling and decreased immune regulatory cytokines. These results support a model of immune dysregulation involving both the innate and adaptive arms of the immune system. Further research is needed to understand the functional role of these markers for those with clinical disease.

## 1 Introduction

Alpha-gal syndrome is an immunoglobulin E (IgE) mediated allergic response to galactose-α-1, 3-galactose (alpha-gal), an epitope expressed in non-primate mammals (1). Individuals who are sensitized to alpha-gal can develop a severe, hypersensitivity reaction after allergen exposure, which is most often caused by consuming mammalian (“red”) meat (2–5). Less common but also reported are reactions to dairy products and other oral, intradermal, and intravenous exposures to alpha-gal (such as infusion of some monoclonal antibodies therapies, antivenom, and products containing gelatin) (6–8).

Alpha-gal syndrome is triggered by the bite of a tick and is typically associated with three-host tick species which demonstrate low host specificity (9). Other sufficient causes might influence development of alpha-gal syndrome, but they are not well understood. We do know that several tick species, including *Amblyomma americanum* (L., 1758), express the alpha-gal antigen (10). Other globally implicated species are: *Ixodes scapularis* (Say, 1821)(United States), *Ixodes ricinus* (L., 1758)(Europe), *Ixodes holocyclus* (Neumann, 1899) and *Ixodes australiensis* (Neumann, 1904)(Australia), *Amblyomma testudinarium* (Koch, 1844) and *Haemaphysalis longicornis* (Neumann, 1901)(Asia), *Rhipicephalus evertsi* (Neumann, 1897) and *Amblyomma hebraeum* (Koch, 1844)(Africa), and *Amblyomma cajennense* (Fabricius, 1787) and *Amblyomma sculptum* (Berlese, 1888)(Central and South America) (11).

Symptomatic individuals with an alpha-gal specific serum IgE of ≥0.1 IU/mL are diagnosed with AGS (7). Symptoms and severity of reactions vary widely, even within a single individual (12). This includes a constellation of gastrointestinal (GI) symptoms including abdominal cramping, nausea, diarrhea, and indigestion (13–15). It can also include more classic symptoms of type 1 reactions such as urticaria, angioedema, and shortness of breath. Isolated GI symptoms can be present in some patients, further confounding diagnosis in the absence of other “classic” allergic symptoms.

Due to the recent emergence of AGS, the delayed onset of allergic symptoms, and the fact that patients may be unaware of a tick-bite exposure, delayed diagnosis is common (16). Activity and behavioral co-factors including exercise, alcohol consumption, and concomitant medications increase reaction frequency and severity (17). Repeated exposure to tick bites can lead to persistent or worsening symptoms and avoidance of tick bite exposures is an important strategy in clinical management of AGS (18). Although AGS is also effectively managed with a patient-specific avoidance diet and emergency medical interventions, it may become life-threatening if not well-controlled and has serious impacts on quality of life.

Landsteiner and Miller first described the alpha-gal epitope during their seminal work on primate serology, noting its similarity to the blood group B-antigen (19–21). In primates, the enzyme responsible for producing alpha-gal is coded by the α-1, 3-galactosyltransferase (*GGTA1*) gene (22). This gene was inactivated 20 to 30 million years ago in catarrhine primates, stopping synthesis of alpha-gal and rendering the molecule immunogenic to humans.

Several theories exist to explain why this inactivation occurred. One posits that multiple endo- and ectoparasites express alpha-gal, so species capable of recognizing alpha-gal as “non-self” may have gained a survival advantage (23, 24). Other theories suggest a singular catastrophic infectious event or a general evolutionary improvement in antibody function drove this change (22). Regardless of the precise cause, the downstream effect is the presence of circulating anti-gal antibodies in humans, rendering the alpha-gal epitope as immunogenic to humans.

It is likely that both the innate and adaptive immune systems contribute to the development of alpha-gal syndrome (AGS) (17). Multiple studies have shown that a T-helper 2 (T_H_2) response is integral to AGS development (25–27). This finding marks a significant departure from previous views that carbohydrates do not induce IgE-mediated allergic responses (28). In the case of alpha-gal, tick bites uniquely trigger a class switch to IgE, which drives the hypersensitivity reactions seen in AGS. This process may be guided by basophil activation, which may trigger mast cell and TNF-α proliferation with cytokines interleukin 4 (IL-4) and interleukin (IL-13) driving the class switch; however, this immunologic mechanism has not been well studied (10).

One of the most compelling theories proposed to explain the clinical heterogeneity observed in AGS is the glycolipid hypothesis (29–31). The delayed onset of symptoms suggests that the time between ingestion and reaction involves key mechanisms which may differ between alpha-gal-containing glycolipids and glycoproteins, which both contain the epitope but are processed differently (32). Digestion of triglycerides, the main form of dietary lipids, and its products take time with peak appearance in the blood hours after ingestion (27).

During lipid digestion, dietary fats are broken down and absorbed by intestinal cells, where they are reassembled and packaged into chylomicrons—lipoprotein particles that enter the bloodstream several hours after a meal. If alpha-gal containing glycolipids are protected within micelles and incorporated into chylomicrons, they may evade digestion and reach systemic circulation intact (33). This delayed transport may explain why alpha-gal allergic reactions are more severe following high-fat meals, as chylomicrons carrying alpha-gal epitopes can trigger basophil or mast cell activation in sensitized individuals. *In vitro* work has shown alpha-gal transport for only cells exposed to digested lipids but not proteins (34). Studies have reported reactions are more severe following consumption of meat cuts with higher fat content (27, 32, 35). Alcohol and exercise have been identified as concomitant factors contributing to more frequent and/or severe reactions; both increase presence of lipids within the bloodstream (8, 36).

Intersubject variability including presence of chronic conditions could also contribute to the severity of clinical presentation (33) or present unmeasured confounding in AGS epidemiology. Sensitization to alpha-gal has also been linked to one of the leading causes of death and disability in the United States—cardiovascular disease (37). In a study that controlled for confounders, stratified by age, and compared multiple IgE-mediated antigens, the presence of alpha-gal –specific IgE was associated with a greater burden of coronary artery atheroma and the presence of unstable plaques (37). Particularly relevant to this study, this association was observed in individuals who were sensitized but did not have alpha-gal syndrome (AGS). Microbiome composition of individuals also may affect the development of AGS (27).

To better understand AGS, mechanisms which trigger class switch to IgE may elucidate biomarkers important to AGS etiology and inform future treatment. In this work, we performed a comparative immunologic analysis using serum samples from well-characterized patient cohorts. By comparing immunological profiles of sensitized and non-sensitized individuals we attempt to delineate the unique immunological signatures of those mounting a primary immune response to alpha-gal versus those who do not have evidence of a primary response. We hypothesized AG+ and AG- groups would exhibit distinct immunologic profiles, including elevated pro-inflammatory and T_H_2-associated cytokines.

## 2 Materials and methods

### 2.1 Study population

Banked sera from the community-based ALL-IN SC study were used for this research. ALL-IN SC was a statewide infectious disease study which recruited participants using convenience sampling; details of the study have been reported elsewhere (38). ALL-IN SC was approved by the University of South Carolina Institutional Review Board (Pro00117562) and the informed consent included an agreement to have samples used for future research studies. Whole blood samples were collected in 4 mL EDTA and/or SST tubes and stored in a portable refrigerator to the main laboratory. The SST tube samples were processed, sera were separated and stored in a –80 °C freezer until serological testing was performed.

### 2.2 Alpha-gal specific IgE testing

To assess alpha-gal prevalence in a cross-sectional manner, serum samples were analyzed by the Commins Laboratory at the University of North Carolina at Chapel Hill. Alpha-gal-specific IgE and total IgE concentrations were measured using commercially available ImmunoCAP assays on the ImmunoCAP 250 instrument (Phadia 250; Thermo Fisher Scientific). Due to limited serum volume from 2022 participants (150μL per sample instead of the recommended 300μL), samples were diluted 1:2. As a result, a threshold of ≥0.2 IU/mL was used to define alpha-gal sensitization for the 2022 cohort. For samples collected in 2023, where sufficient serum volume was available, individuals were considered sensitized if alpha-gal-specific IgE was ≥0.1 IU/mL. Across all participants, sensitization status was classified as either positive or negative based on these cohort-specific cutoffs—participants were asked about clinical symptoms, but none of the participants with detectable titers reported AGS compatible disease (18), therefor classifying all persons as sensitized.

### 2.3 Immunological profiling

We used two Bio-Plex Pro (BioRad^®^) assays to examine 46 analytes from the tumor necrosis factor (TNF) superfamily, pro-inflammatory cytokines, and cytokines involved in T_H_2 immune responses: BioRad’s Bio-Plex Pro Human Inflammation Panel 1, 37-Plex (catalog #171AL001M) and a customized BioRad’s Bio-Plex Pro Human Cytokine 9-plex Assay. Testing was performed using two different assays; the first assay looked at 37 analytes and the second looked at 9 targets. A total 111 patients were analyzed for this research (n=111); all patients had sera available for the 37-plex human cytokine assay but only 76/111 people had sera available for the 9-plex testing.

Following manufacturer instructions, standards were incorporated to calculate standard curves based on the fluorescence intensity, along with two quality controls to confirm the analysis was performed correctly. All samples were diluted 1/4 of their concentration, and all the final concentrations were calculated automatically by the Bio-Plex Manager Software (Bio-Rad Laboratories, Hercules, CA). For statistical analysis, values with out-of-range fluorescence readings were manually calculated approximating the standard curve for each plate and analyte following a five-parameter logistic equation:


$$FI=A+\frac{\left(B-A\right)}{{\left(1+{\left(\frac{Conc}{C}\right)}^{D}\right)}^{E}}$$

where A is the background fluorescence (estimated fluorescence at concentration 0), B is estimated maximum fluorescence, C is midrange concentration, D is the slope of the tangent at midpoint, and E is an asymmetry factor.

### 2.4 Statistical analysis

There were 50 AGS+ individuals and these participants were matched by gender, age, and race/ethnicity using propensity scoring to AGS- individuals; sera were available for 111 of these participants. After alpha-gal status was determined, univariate analysis was performed to compare median concentrations of all analytes and to confirm no significant differences for matched variables (**Table 1**). Participants who reported chronic disease concerns, or a positive family history for chronic diseases were also analyzed in this research.

**Table 1:** Demographics, tick bite exposure, and chronic conditions by alpha-gal status.

Variable	All participants n=111	Non-sensitized n (%) n=61	Alpha-gal sensitized n (%) n=50	p-value
Median age	55	56	53.4	
Race/ethnicity				0.49
Non-Hispanic White	21 (19.27)	14 (12.84)	7 (6.42)	
Non-Hispanic Black	30 (27.52)	15 (13.76)	15 (13.76)	
Multiracial/Other	58 (53.21)	32 (29.36)	26 (23.85)	
Gender				0.98
Male	62 (55.86)	34 (30.63)	28 (25.23)	
Female	49 (44.14)	27 (24.32)	22 (19.82)	
How often do you find ticks biting you?				0.07
Never	79 (73.15)	49 (45.37)	30 (27.78)	
Rarely	24 (22.22)	9 (8.33)	15 (13.89)	
Sometimes/Often	5 (4.63)	2 (1.85)	3 (2.78)	
Arthritis				0.12
Yes	31 (28.44)	21 (19.27)	10 (9.17)	
No	78 (71.56)	40 (36.70)	38 (34.86)	
Asthma				0.32
Yes	18 (16.51)	12 (11.01)	6 (5.50)	
No	91 (83.49)	49 (44.95)	42 (38.53)	
Cancer				0.84
Yes	24 (22.02)	13 (11.93)	11 (10.09)	
No	85 (77.98)	48 (44.04)	37 (33.94)	
Cognitive impairment				1.00
Yes	10 (9.17)	6 (5.50)	4 (3.67)	
No	99 (90.83)	55 (50.46)	44 (40.37)	
Diabetes				0.01
Yes	37 (33.94)	27 (24.77)	10 (9.17)	
No	72 (66.06)	34 (31.19)	38 (34.86)	
High cholesterol				0.02
Yes	31 (28.44)	23 (21.10)	8 (7.34)	
No	78 (71.56)	38 (34.86)	40 (36.70)	
Heart disease				0.05
Yes	23 (21.10)	17 (15.60)	6 (5.50)	
No	86 (78.90)	44 (40.37)	42 (38.53)	
Hypertension				0.51
Yes	47 (43.12)	28 (25.69)	19 (17.53)	
No	62 (56.88)	33 (30.28)	29 (26.61)	
Stroke				0.50
Yes	10 (9.17)	7 (6.42)	3 (2.75)	
No	99 (90.83)	54 (49.54)	45 (41.28)	

Shapiro-Wilk test for normality was performed on all cytokines, and each yielded non-normal distributions (p < 0.05). Univariate logistic regression was performed to assess initial associations, with those yielding p-value ≤ 0.30 examined for multi-collinearity, Variables with VIF values >10 were removed, with remaining variables then evaluated using stepwise selection and significant variables. A Benjamini-Hochberg correction was performed for multiple testing. All analyses were performed in SAS On-Demand, SAS V9.4 (SAS Institute Inc., Cary, NC) and STATA SE v18 (STATA Corp LLC, College Station, TX).

## 3 Results

### 3.1 Participant characteristics

A total of 111 participants were included in the analysis, of whom 50 (45.05%) were alpha-gal sensitized (AGS+) and 61 (54.95%) were not sensitized (AGS−). Because of sera availability and propensity score matching methods, we had more controls than cases. We elected not to drop any controls to avoid biased selection of controls. Most participants identified as multiracial or other (53.2%), followed by non-Hispanic Black (27.5%) and non-Hispanic White (19.3%). Gender was evenly distributed, with 56% identifying as male and 44% as female. There were no statistically significant differences by alpha-gal sensitization status by age, race/ethnicity, or gender (**Table 1**).

Tick exposure history was marginally associated with alpha-gal sensitization (p = 0.07). Among sensitized individuals, 13.9% reported rarely finding ticks biting them, compared to 8.3% of non-sensitized participants. Chronic health conditions including arthritis, asthma, cancer, cognitive impairment, hypertension, and stroke were not significantly associated with sensitization (all p > 0.05). However, a significantly lower proportion of alpha-gal positive individuals reported having diabetes (p = 0.01), high cholesterol (p = 0.02), and heart disease (p = 0.05) compared to alpha-gal negative participants.

### 3.2 Univariate regression model of molecular analytes

Forty-six analytes across five immunologic categories were analyzed for potential differences between groups: 19 targets were statistically significant on univariate regression **Table 2**). Total IgE and alpha-gal specific IgE was higher among the sensitized group (p < 0.001). Four innate immune response analytes were statistically significant, with CD163 (p < 0.001) and IL-12p40 (p = 0.023) concentrations being moderately lower in the sensitized group than the non-sensitized group, and IFNα2 (p < 0.001) substantially lower in the sensitized group. However, pentraxin (p < 0.001) was moderately higher in the sensitized group. Among type two allergic response analytes, two were statistically significant with inverse associations: chitinase (p < 0.001) was moderately lower in the sensitized group, whereas CD30 (p < 0.001) concentrations were substantially higher in the sensitized group.

**Table 2:** Median concentrations and univariate logistic regression outcome for human cytokine assays by immunologic functional categories*.

Immune function category and targets	Median (IQR) non-sensitized (pg/ml) N=61	Median (IQR) Alpha-Gal Sensitized (pg/ml) N=50	p-value
Alpha-gal specific analytes			
sIgE	**0.01** (0.00 - 0.01)	**0.45** (0.24 - 1.13)	.
Total IgE	**93.80** (36.10 - 248.00)	**837.00** (268.00 - 2559.00)	<0.001
Innate immune response			
CD163	**15160.29** (6949.82 - 32163.22)	**155242.69** (7496.48 - 279558.19)	<0.001
IFNα2	**189.52** (143.10 - 272.72)	**3.10** (3.10 - 90.41)	<0.001
IFNβ	**15.59** (13.36 - 19.95)	**0.90** (0.90 - 13.92)	0.584
IL-12p40	**5.50** (5.50 - 5.50)	**5.50** (5.50 - 5.50)	0.023
IL-12p70	**1.30** (1.30 - 1.30)	**1.30** (1.30 - 1.30)	.
IL-27	**221.20** (194.95 - 247.66)	**3.80** (3.80 - 208.07)	0.801
IL-28	**10.70** (10.70 - 10.70)	**10.70** (10.70 - 10.70)	.
IL-29	**5.20** (5.20 - 5.20)	**5.20** (5.20 - 5.20)	0.394
IL-34	**61.90** (61.90 - 61.90)	**61.90** (61.90-61.90)	0.182
Pentraxin	**3729.47** (1811.39 - 5758.15)	**8719.30** (2942.67 - 16368.41)	<0.001
Type 2/allergic immune response			
Chitinase	**2903.57** (1861.12 - 4016.68)	**14260.46** (2556.14 - 21298.48)	<0.001
CD30	**495.32** (278.31 - 1070.87)	**5456.58** (456.70 - 7874.17)	<0.001
IL-4*	**0.19** (0.19 - 0.19)	**0.19** (0.19 - 0.19)	0.300
IL-5*	**7.90** (3.63 - 11.55)	**5.43** (3.63- 10.95)	0.740
IL-9*	**3.62** (3.62 - 3.62)	**3.62** (3.62 - 3.62)	0.511
IL-13*	**1.93** (1.22 - 3.43)	**0.97** (0.31 - 3.84)	0.493
IL-19	**8.50** (8.50 -8.50)	**8.50** (8.50 -8.50)	0.054
TSLP	**23.87** (14.73 - 32.58)	**3.00** (3.00 – 14.73)	0.229
Pro-inflammatory immune response			
gp130	**12334.79** (8028.12 - 21348.04)	**93478.17** (12627.66 - 154798.00)	<0.001
IFNγ	**6.30** (6.30- 6.30)	**6.30** (6.30- 6.30)	.
IL-1α*	**3.85** (1.99 - 5.70)	**3.85** (2.92 - 5.70)	0.151
IL-1β*	**0.46** (0.29 - 0.71)	**0.58** (0.29 - 0.96)	0.251
IL-6Ra	**2442.44** (797.51 - 4778.03)	**10134.00** (1310.02 - 14449.52)	<0.001
IL-8	**4.50** (4.50 - 67.39)	**4.50** (4.50 - 285.53)	0.026
IL-11	**0.10** (0.10 - 0.10)	**0.10** (0.10 - 0.10)	.
IL-17A*	**7.80** (6.57 - 8.63)	**7.80** (6.98 - 9.47)	0.255
IL-20	**2.70** (2.70 - 2.70)	**2.70** (2.70 - 2.70)	0.113
IL-22	**5.30** (5.30 - 5.30)	**5.30** (5.30 - 5.30)	0.479
IL-26	**8.20** (8.20 -8.20)	**8.20** (8.20 -8.20)	.
IL-32	**3.90** (3.90 - 3.90)	**3.90** (3.90 - 1803.87)	<0.001
MMP-1	**106.80** (106.80 - 106.80)	**106.80** (106.80 - 106.80)	0.910
MMP-2	**5369.16** (1224.50 - 10866.50)	**84912.50** (5369.16 - 109278.12)	<0.001
MMP-3	**395.70** (395.70 - 395.70)	**395.70** (395.70 - 395.70)	0.015
Osteopontin	**9702.03** (7939.43 - 11932.20)	**37679.19** (9789.09 - 54618.55)	<0.001
TNFα*	**10.38** (7.64 - 12.43)	**12.09** (7.64 - 15.82)	0.28
TNFR1	**786.73** (419.27 - 1092.93)	**5136.29** (576.79 - 7509.84)	<0.001
TNFR2	**311.26** (154.17 - 475.44)	**1462.71** (240.96 - 2074.84)	<0.001
TWEAK	**203.63** (155.89 - 280.90)	**1717.48** (155.89 - 2560.38)	<0.001
B-cell and immune-regulatory response			
APRIL	**3440.10** (3440.10 - 69889.16)	**3440.10** (3440.10 - 3440.10)	0.585
BAFF	**3812.65** (2187.50 - 12473.65)	**22866.52** (467.00 - 38685.98)	<0.001
IL-1Rα*	**289.05** (163.83 - 471.93)	**357.99** (184.21 - 523.29)	0.514
IL-2	**1.20** (1.20 - 1.20)	**1.20** (1.20 - 1.20)	.
IL-10	**1.70** (1.70 -1.70)	**1.70** (1.70 -1.70)	0.188
IL-35	**171.57** (104.81 - 280.36)	**20.60** (20.60 - 20.60)	<0.001
LIGHT	**43.36** (21.16 - 59.05)	**14.80** (14.80 - 17.98)	<0.001

*performed on subset of patient sera with remaining sera, non-sensitized group n=36 and sensitized group n=40. Bold values are the median concentration for each analyte.

Nine pro-inflammatory analytes demonstrated significant associations with sensitization—all were positively associated with AGS. Gp130 (p < 0.001), IL-8 (p = 0.026), IL-32 (p < 0.001), and MMP-3 (p < 0.015) concentrations were moderately higher in the sensitized group, and MMP-2 (p < 0.001), TNFR1 (p < 0.001), TNFR2 (p < 0.001), and TWEAK (p < 0.001) were all substantially higher in the sensitized group. Three regulatory analytes, for B-cell or other immune pathways, yielded significant associations with AGS. BAFF demonstrated substantially higher concentrations in the sensitized group, whereas LIGHT (a.k.a. TNFSF14) (p < 0.001) and IL-35 (p < 0.001) demonstrated moderately lower and substantially lower concentrations, respectively.

### 3.3 Multivariable model of molecular analytes

Logistic regression with stepwise selection was used to identify cytokines most strongly associated with alpha-gal sensitization. After accounting for multicollinearity and correcting for multiple testing using the Benjamini-Hochberg procedure, three cytokines were independently associated with sensitization status **Table 3**): higher concentrations of IL-1α were associated with increased odds of AGS (OR = 1.38, 95% CI: 1.01 -1.90, p = 0.045). Lower median concentration of IL-35 (OR = 0.98, 95% CI: 0.97 – 0.99, p < 0.001), LIGHT (OR = 0.94, 95% CI: 0.89 – 0.99, p = 0.012) and IFN- α2 (OR = 0.99 (0.98 – 0.99), p = 0.041) were associated with sensitization. These targets represent a mix of innate (IFN- α2), pro-inflammatory (IL-1α) and regulatory (IL-35 and LIGHT) immune pathways and highlight dysregulated immune balance among sensitized individuals. Linear regression fitted lines with 95% confidence intervals over scatter plots for each analyte are displayed in **Figures 1A–D**.

**Table 3:** Multivariable logistic regression model of probability of alpha-gal sensitization.

Analyte	p-value	OR	95% CI
IFNα2	0.041	0.99	0.98 - 0.99
IL-1α*	0.045	1.38	1.01 - 1.90
LIGHT	0.012	0.94	0.89 - 0.99
IL-35	0.006	0.98	0.97 - 0.99

**Figure 1:**
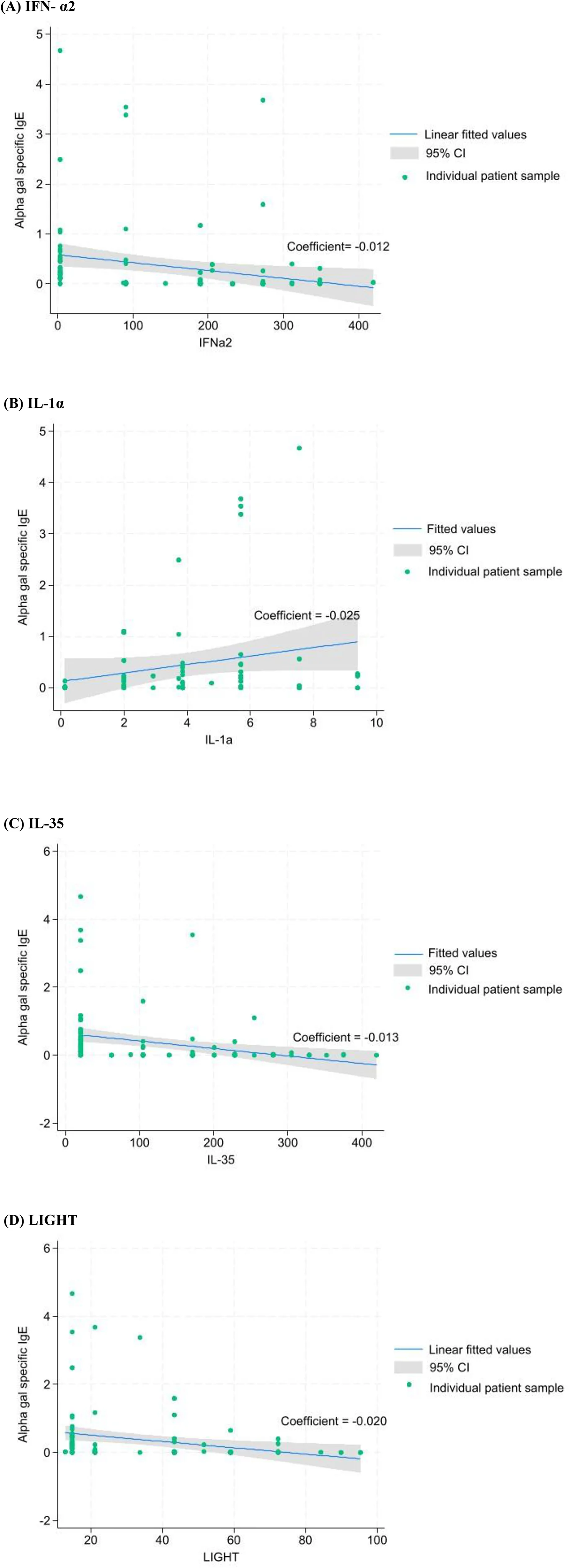
Linear regression fitted lines with 95% confidence intervals over scatter plots for all variables statistically significant on the multivariable model: **(A)** IFN- α2, **(B)** IL-1α, **(C)** IL-35, and **(D)** LIGHT*.

## 4 Discussion

Alpha-gal syndrome presents a novel and complex allergic phenotype driven by sensitization to a carbohydrate epitope rather than a protein, a notable departure from classical IgE-mediated allergies. The immune mechanisms underlying AGS are only partially understood, and our study contributes to this growing body of knowledge by identifying distinct immunologic signatures associated with alpha-gal sensitization versus non-sensitized persons in a community-based cohort.

Our results support the hypothesis that alpha-gal sensitization is characterized by a dysregulated immune response that includes both pro-inflammatory and impaired regulatory signaling. However, we may not be able to extend this finding to AGS since this study only examined sensitized, non-symptomatic individuals. We identified IL-1α, IFNα2, IL-35, and LIGHT (TNFSF14) as the strongest independent predictors of alpha-gal sensitization, even after adjusting for multiple testing. Elevated IL-1α levels were associated with greater odds of sensitization, consistent with its role as an early mediator of epithelial inflammation and innate immune activation (39). In contrast, lower IFNα2 levels, a type I interferon, were associated with sensitization, reflecting its potential role attempting to stop or slow down a Th2 mediated IgE response (40, 41). Similarly, reduced levels of LIGHT concentrations may reflect reduced TNF-superfamily–mediated inflammatory or T-cell costimulatory activity among sensitized patients (42, 43). In contrast, reduced levels of IL-35 suggest impaired immunoregulatory control mechanisms among sensitized individuals (44, 45).

The reduced concentrations of IL-35 and LIGHT in sensitized participants support existing models of allergic disease where loss of immunologic tolerance contributes to chronic sensitization. Lower levels of IL-35, an anti-inflammatory cytokine, could represent sensitized patient’s lack of inflammatory immunoregulation leading to systemic inflammation (45). LIGHT, although capable of promoting inflammation in some contexts, also plays a modulatory role in T cell homeostasis (46). In murine models, overexpression of LIGHT results in enhanced T_H_1 cytokine production and intestinal inflammation, yet in our human data, decreased LIGHT levels were associated with alpha-gal sensitivity (47, 48). Given AGS is thought to induce a T_H_2 response (26), lower LIGHT levels could reflect the body’s shift away from T_H_1 cytokine production towards a T_H_2 mediated response in sensitized individuals. This apparent paradox highlights the context-specific role of LIGHT and suggests that its regulation—rather than its absolute concentration—may be more relevant in allergic phenotypes like AGS.

While we only found four immune targets statistically significant on the multivariate model, several analytes were statistically significant by univariate regression. Given the low sample size, which potentially lowered power, looking at the potential role these additional analytes play in the overall alpha-gal sensitization pathway is worth cautious discussion. Several innate immune markers, including CD163, Chitinase-3-like 1, and gp130, were elevated, indicating sustained innate activation. In parallel, we observed lower levels of interferons (IFNβ), IL-10, and IL-27, which are commonly associated with anti-viral defense and immune regulation (49–51). This pattern suggests a failure in the regulatory “braking system” that would normally counterbalance innate and T_H_2-skewed inflammation. Interestingly, concentration of cytokines of major importance in IgE-mediated allergic reactions such as IL-4 and IL-13 did not differ between groups. However, our cohort of sensitized persons represent those with laboratory evidence of sensitization without clinical symptoms. The sensitized group’s mostly low alpha-gal specific IgE levels and contrasting relatively high total IgE levels in the context of low tick bite frequency may be reflective of other immunologic processes (e.g. eczema, parasitic infection)—the precise correlation of immunologic analyte levels to veritable tick-mediated IgE alpha-gal allergy cannot be confirmed with the current methods. Large studies enrolling healthy controls, sensitized/non-symptomatic, and symptomatic participants are needed to better understand these differences between phenotypes.

Our findings could offer mechanistic insight into the unusual clinical presentation of AGS. Unlike typical IgE-mediated food allergies, AGS reactions are delayed by several hours and are often associated with high-fat meals. This delay may reflect differences in antigen processing: as previously discussed, alpha-gal–containing glycolipids are transported via chylomicrons, which enter the bloodstream hours after ingestion (11). This lipid-associated transport pathway may not only explain the delayed hypersensitivity reactions but could also influence the strength and type of immune activation. Glycolipid presentation via CD1 molecules to NKT cells, rather than via MHC-II to conventional T cells, may favor T_H_2 polarization and support isotype switching to IgE—a hallmark of AGS clinical presentation (52). Our findings support the potential immunologic shift away from a T_H_1 response in favor of a T_H_2 one, specifically that higher CD30 and IL-19 levels among sensitized groups reflect a T_H_2 associated profile (53, 54). While the following analytes were not statistically significant on univariate regression, IL-4, IL-5, IL-9 and IL-13 all had higher average concentrations in the sensitized group, suggesting potential T_H_2 polarization (55, 56).

The difference in prevalence of chronic disease by alpha-gal sensitization, while interesting, should be interpreted with caution. These associations may reflect unmeasured confounding, differential tick exposure patterns, or myriad metabolic differences that influence lipid digestion and immune activation. The findings are interesting as only chronic diseases with previous association with alpha-gal and biological plausibility showed significance. For example, altered postprandial lipid metabolism in individuals with diabetes may affect how glycolipid antigens are absorbed and presented (57), potentially modulating downstream immune responses and higher rates of cardiovascular disease are observed in those with alpha-gal sensitization.

This study has some limitations worth discussing. First, the sample size limited stratified analysis and model complexity. Second, the use of stored sera may have introduced degradation of certain analytes, although this would bias toward the null and samples were relatively recent (only stored at -80C for one to two years prior to analysis). Third, the cross-sectional design prevents us from determining the temporality or causality of observed cytokine differences and this data allowed us to observe differences between sensitized and non-sensitized individuals. Fourth, this study did not include AGS symptomatic patients, limiting the clinical utility of our findings and shifting the findings’ value towards a more academic-focused audience. An important piece of the alpha-gal puzzle is understanding the immunologic mechanisms which push individuals into syndromic manifestations from alpha-gal exposure, which were not able to be studied in this cross-sectional study sample.

In conclusion, our data reveals immunologic profiles that distinguish individuals with alpha-gal sensitization from their non-sensitized peers. These profiles include increased pro-inflammatory signaling and reduced immune regulatory cytokines, consistent with a model of persistent immune activation and impaired tolerance. Our findings support the theory that both the innate and adaptive arms of the immune system are involved in AGS pathogenesis and suggest potential biomarkers for future research. Elucidating the cellular and molecular pathways of alpha-gal sensitization holds promise for developing targeted therapies for this emerging condition, situated at the crossroads of parasite–host biology and host immunity.

## Data Availability

The raw data supporting the conclusions of this article will be made available by the authors, without undue reservation.
